# Consumer Preferences and Acceptance of Meat Products

**DOI:** 10.3390/foods9060708

**Published:** 2020-06-01

**Authors:** Andrea Garmyn

**Affiliations:** Department of Animal and Food Sciences, Texas Tech University, Lubbock, TX 79409, USA; agarmyn@gmail.com; Tel.: +1-785-410-4618

At the point of purchase, consumers often use extrinsic cues such as color, marbling, leanness, packaging, and price to determine which meat product(s) to buy. The value placed on such cues may vary regionally or even be influenced by the demographic characteristics of the consumer. Tenderness, juiciness, and flavor remain the three pillars of cooked meat palatability, all linked to consumer satisfaction. Historically, tenderness has been the single most important factor affecting beef palatability, yet previous work has shown that flavor becomes the most important aspect of eating satisfaction when tenderness is acceptable. Consumers can distinguish marbling and consequently flavor differences in some muscles, and are willing to pay premiums for the type of flavor they prefer. Several consumer studies over the past two decades have collectively shown that consumer overall acceptability ratings are more highly correlated with flavor ratings than tenderness or juiciness ratings in beef and lamb. However, the role of flavor in the acceptability of muscles from primals other than the rib or loin is not entirely clear. Moreover, consumer acceptance of and preference for flavor potentially shifts when dealing with value-added or processed meats as opposed to fresh meats. This Special Issue includes 12 valuable scientific contributions, including one review article and 11 original research articles, focusing on antemortem and postmortem factors that influence the sensory acceptability of meat products across the major red meat species of beef, lamb, and pork. 

Payne et al. [1] investigated the influence of lamb age class on Australian consumer eating quality scores of eight lamb cuts. Their results showed little difference in the eating quality between “new season” (approx. 5–8 months old) and “old season” (approx. 10–12 months old) lamb, highlighting the market potential for old season lamb products at retail. 

Five of the original research articles focused on the influence of postmortem factors, such an enhancement [2,3] or incorporation of non-meat ingredients [4], postmortem aging [5], and packaging [6] on consumer eating quality. Lees et al. [2] assessed the impact of kiwifruit extract (actinidin) on consumer sensory outcomes for beef strip loin and outside flat. In addition, cooking method (grill or roast) and postmortem aging length (10 or 28 days) were also tested. Kiwifruit extract improved all palatability traits (tenderness, juiciness, flavor liking), resulting in greater overall liking for both strip loins and outside flats. Lees et al. [2] suggested actinidin infusion provides an opportunity to improve eating experiences for beef consumers. In another enhancement study, Garmyn et al. [3] explored the eating quality of several beef muscles cooked and served as fajita meat strips after enhancement with either a phosphate-based marinade or a “clean label” alternative ingredient (sodium bicarbonate). Those muscles included the outside skirt, inside skirt, flank, inside round cap, and bottom sirloin flap. Enhanced samples were scored more favorably than non-enhanced samples for all palatability traits; samples enhanced with sodium bicarbonate were more tender and juicier than samples enhanced with phosphate. According to consumers, the inside round cap was the least suitable option for preparation as fajitas. However, creating a “clean label” enhanced fajita product was possible without compromising cooking yield or consumer satisfaction [3]. 

Consumer response to reformulated burger patties with ingredients that could improve healthfulness [4] is another topic area in this Special Issue. Taylor et al. [4] tested various levels of tempeh inclusion (10%, 20%, and 30%) in beef patties. Their sensory experiments revealed that beef patties could include up to 10% tempeh; however, consumers rated visual appearance lower, along with less flavor and overall acceptability when patties included 20% to 30% tempeh [4]. 

The final two research articles focusing on postmortem factors that influence consumer eating quality dealt with extended postmortem aging of beef [5] and modified atmosphere packaging of pork [6]. Garmyn et al. [5] investigated the effect of extending the postmortem aging of beef strip loins from 21 to 84 days. Based on their results, samples should not be wet-aged longer than 63 days to prevent negative eating experiences for consumers; however, storage conditions (i.e., temperature) could potentially be adjusted to accommodate longer chilled storage without compromising flavor and overall palatability to the same extent [5]. Peng et al. [6] investigated the effects of high oxygen modified atmosphere packaging (MAP) of pork loins compared to vacuum packaging on eating quality and color following presentation in simulated retail display conditions. Ultimately, retailers should consider vacuum packing the preferred option over high-oxygen MAP, given the inferior consumer acceptability for palatability traits and greater lipid oxidation of MAP samples.

Several research articles in this Special Issue involved the investigation of the consumers’ backgrounds and how cultural differences could influence sensory perception and acceptance of meat products. Mena et al. [7] explored the meal and snacking behavior of older adults in Australia and China. In this study, demographics influenced consumer preferences towards food, as older consumers in China and Australia differed in their responses to product traits and segmentation. In another cross-cultural study investigating red meat eating quality, Hastie et al. [8] used a mixed method approach involving both perceptual mapping (qualitative) and sensory (quantitative) methodologies to gain consumer sensory insights into sheep meat and beef. Australian and Asian consumers differed in their perception of ‘premiumness’ of meat products, which could be related to the traditional meat preparation and presentation styles between those groups of consumers. Moreover, demographic factors, specifically age, influenced eating quality and willingness to pay for sheepmeat and beef. O’Reilly et al. [9] wanted to determine whether demographic factors influenced consumer perceptions of sheep meat eating quality. Their results show consumer age, gender, household size, and income influenced sensory scores, but the impact varied across the three countries where testing took place—Australia, China, and United States. Frequency of lamb consumption is also a relevant factor when assessing eating quality, but again varied between the three countries [9].

Felderhoff et al. [10] aimed to quantify the relative contribution of palatability traits (tenderness, juiciness, and flavor) to beef satisfaction and assess if and to what extent certain demographic variables influence satisfaction. The authors found that flavor was the largest contributor to satisfaction in comparison to tenderness or juiciness, accounting for 59% of the overall rating. The results also indicate that age, income, and gender influenced satisfaction [11].

Arenas de Moreno [11] conducted surveys in three regions of Venezuela to determine buying expectations, motivations, needs, perceptions, and preferences of beef consumers, and their acceptance of domestic and foreign beef. Their results show that two factors explain 74% of the common variance in beef consumption. The first factor focuses on intrinsic factors, such as color, smell, tenderness, flavor, juiciness, and freshness, while the second factor involves more extrinsic factors, primarily product origin. The authors hope to use these results to design and implement strategies to recover and enhance the domestic beef demand in Venezuela [11]. 

Finally, Miller [12] reviewed the drivers of consumer liking for beef, pork, and lamb, suggesting the drivers are interrelated across species, but differences exist. For example, animal age, animal diet, and marbling influence consumer liking across species. For beef, tenderness has historically been the main driver of consumer liking, but as tenderness has improved and tenderness variation has been reduced, flavor has become a greater determinant to overall liking. Flavor, which is influenced by a number of antemortem and postmortem factors, was explored along with tenderness and juiciness, to determine how changes in palatability traits in response to those factors influence overall liking. Drivers of pork consumer liking can be influenced by pH, color, water holding capacity, animal diet, and presence of boar taint compounds. For lamb, the flavor, which is typically a direct reflection of animal diet and animal age, continues to be the primary driver of consumer liking; however, cultural differences and preferences may exist due to the variable consumption rates in certain countries. 

In summary, the Special Issue “Consumer Preferences and Acceptance of Meat Products” demonstrates that the value of different palatability traits has evolved over time. Moreover, consumer acceptance and preference are not solely determined by the inputs of the meat itself, but can also be influenced by various demographic factors. In addition, consumers’ views of meat products vary regionally and vary by species. 
